# Various Physiological Response to Graphene Oxide and Amine-Functionalized Graphene Oxide in Wheat (*Triticum aestivum*)

**DOI:** 10.3390/molecules23051104

**Published:** 2018-05-07

**Authors:** Juanni Chen, Liang Yang, Shili Li, Wei Ding

**Affiliations:** Laboratory of Natural Product Pesticide, College of Plant protection, Southwest University, Chongqing 400715, China; jemmy@swu.edu.cn (J.C.); ylwzling@163.com (L.Y.); lsl203lst@163.com (S.L.)

**Keywords:** graphene oxide, amined-functioned graphene oxide, *Triticum aestivum*, phytotoxicity

## Abstract

An increasing number of investigations have been performed on the phytotoxicity of carbon-based nanomaterials duo to their extensive use in various fields. In the present study, we investigated the phytotoxicity of unfunctionalized graphene oxide (GO) and amine-functionalized graphene oxide (G-NH_2_) on wheat (*Triticum aestivum*) in the concentration range from 125 to 2000 μg/mL after 9 days of hydroponic culture. Our results found that the incubation with both nanomaterials did not affect the final seed germination rate, despite some influence in the initial stage. Transmission electron microscopy (TEM) observations indicated that exposure to GO at a high concentration (above 1000 μg/mL) resulted in a severe loss of morphology of seedlings, and a decrease in root length, shoot length and relative biomass, along with obvious damage to plant tissue structures (root, stem and leaf) when compared with the control. GO induced increased damage to root cells, which were determined by electrolyte leakage. Conversely, the plant growth was enhanced under G-NH_2_ exposure, and the root and stem lengths were increased by 19.27% and 19.61% at 2000 μg/mL, respectively. The plant tissue structures were not affected, and neither GO nor G-NH_2_ were observed to accumulate in the wheat plant root cells. The present investigations provide important information for evaluation of the environmental safety of GO and better understanding plant-nanoparticle interactions.

## 1. Introduction

Carbon-based nanomaterials, one of the most attractive nanomaterials with various forms, encompassing fullerenes, single- and multiple-walled carbon nanotubes, carbon nanoparticles, graphene, and so forth, are at the leading edge of the rapidly developing field of nanotechnology [1]. With nano-sized and unique physicochemical properties, carbon-based nanomaterials cumulatively play a role in industrial production, water treatment, food and agriculture field and have inspired an increasing interest in exploring the application of nanoparticles as delivery systems and imaging agents for plant cells and plants [2,3]. Among them, graphene represents the most widely used carbon-based nanomaterial duo to its fascinating physicochemical properties, such as good thermal stability, high surface area, exceptional physiochemical properties, high electronic conductivity and excellent mechanical strength [4]. In accordance with their outstanding properties, graphene and its derivatives have been explored in a variety of commercial applications such as electronic and photonic devices, clean energy, material and biosensors [5,6]. It has been increasingly recognized by researchers that, in addition to the development of novel nanomaterials, parallel efforts should be made to investigate and understand their potential health and environmental effects, given the fact that a substantial use of nanoparticles in various fields has made possible their incidental and direct release and transfer into the soil and atmospheric environment. Plants are an essential base component of all ecosystems and play a critical role in the fate and transport of nanoparticles in the environment through plant uptake and bioaccumulation [7]. Therefore, understanding the physiological effects of nanoparticles is essential.

As more and more studies are performed on nanotoxicity, the phytotoxicity of carbon-based nanoparticles on higher plants has received an increasing controversial attention duo to different findings. From another point of view, it can be noted that the toxic effects vary greatly with different nanomaterial types, plant species, growth stages, concentration, exposure time and surface structure. Among the studies available, Khodakovskaya et al. have reported that multi-walled carbon tubes (MWCNTs) dramatically enhanced the seed germination and growth of crop plants (barley, soybean, tomato and corn), because the CNTs are able to penetrate the thick seed coat and support water uptake inside seeds [8,9]. A different observation was demonstrated by Gajanan Ghodake et al. that MWCNTs were non-hazardous to the seed germination of *Brassica juncea* (*B. juncea*) and *Phaseolus mungo* at the same concentration while enhanced the root growth of *B. juncea* [10]. Another study has shown that SWCNTs promoted the growth of onion and cucumber but strongly inhibited the root elongation of tomato, cabbage, carrot and lettuce [11].

To date, graphene and its derivatives have been proved to toxic to a variety of species, including vertebrates [12], algae [13], bacteria [14,15] and fungi [16]. However, several studies are focused on the effect on plant species, which are extremely sensitive to their growth environment [17]. For example, one study was performed to evaluate the effect of GO on tomato seed germination and seedling growth. Graphene was found to penetrate seed husks that might facilitate water uptake, resulting in faster germination and higher germination rates [18]. Recently, totally contrary results have been reported by Parvin Begum and co-workers who stated that at a higher concentration range from 500 to 2000 mg/L, graphene significantly inhibited plant growth and biomass of cabbage, tomato, and red spinach except lettuce [19]. A similar toxicity effect was also displayed on the red spinach when exposed to 0–1000 mg/L of MWCNTs after 15 days of hydroponic culture, exhibiting growth inhibition and cell death [20]. Unexpectedly, hydrated graphene ribbon (HGR) promoted aged (two years) wheat seed germination and enhanced resistance to oxidative stress, which was connected with further upregulation of the carbohydrate, amino acid, and fatty acids metabolism that determined secondary metabolism [21].

Meanwhile, it is noticed that surface chemistry exerts a significant role in the toxicity effects of carbon-based nanomaterials, especially for CNTs. Functionalized CNTs demonstrated different toxic behaviors but were generally less toxic than non-functionalized CNTs [11]. Alimohammadi M et al. investigated the phytotoxicity to tomato plants by decorating the CNTs with quantum dot and found that the negatively charged nanotubes seemed to have a stronger effect on plant development, showing obvious symptoms of toxicity compared with the positive effects of p-CNTs [22]. A study performed by Villagarcia’s group showed that, when compared with MWCNTs, carboxylated MWCNTs with much higher negative surface charge and dispersion induced a more significant increase in the fresh biomass of tomato [23]. However, by evaluating two types of raw MWCNTs and carboxylated MWCNTs in a laboratory experiment, a recent investigation found that both types of CNTs inhibited seed germination. At the same time, soil supplementation with carboxylated MWCNTs extremely reduced the dry yield of common meadow grass and MWCNTs significantly reduced dry matter yield in all studied grasses [24]. Interestingly, functionalized CNTs in a wide range of concentrations (9–1750 μg/mL) were found to have no effect on the physiology and development of cabbage and carrot [11]. However, the comparison impact of GO and its derivatives on high plants has scantly been examined in the current literature.

Hence, in this work, we conducted a preliminary study to determine the interactions of graphene oxide (GO) and amine-modified graphene (G-NH_2_) with *Triticum aestivum* (*T. aestivum*). Particular efforts were focused on how the nanomaterials influence the growth of wheat seedlings under hydroponic culture conditions and whether GO and G-NH_2_ were accumulated and located in wheat plant root organs. Phytotoxicity studies demonstrated that GO showed negative effects on the growth of seedlings such as root cell death. Contrarily, G-NH_2_ could enhance the extension of root and stem in the present study.

## 2. Materials and Methods

### 2.1. GO and G-NH_2_ Preparation

GO was prepared from natural graphite powders by the modified Hummers method [25]. Firstly, the nature graphite powders (99.99%; Sigma-Aldrich, Saint Louis, MO, USA) were oxidized to produce graphite oxide (GtO). After removing chemical residues by washing with deionized water, the produced GtO dispersion was sonicated (Elamsonic, S60H) for 2 h and exfoliated to obtain the GO sheets. G-NH_2_ was prepared as previously described [26]. GO sheets (1 g) were stirred in a tube containing SOCl_2_ and dimethylformamide (DMF), filtered through a PTFE membrane (pore size 0.2 μm), and then washed with dry methylene chloride to form graphene-COCl. The product was dissolved in a mixture of sodiumazide (1.5 mM) and DMF at room temperature for 40 h. After reaction, the mixture was filtrated to isolate the product, and then sonicated in concentrated hydrochloric acid to yield the amine-modified graphene product. Finally, the product was washed repeatedly with deionized water until the pH of the filtrate was neutral.

The two types of materials were characterized by several techniques. 100 μg/mL of dispersions were prepared, respectively. The particle sizes of GO and G-NH_2_ dispersions were evaluated with dynamic light scattering (DLS) on Malvern Zetasizer Nanoseriesa (Malvern, UK). Then, the pH values of dispersions were adjusted ranging from 2.0 to 10.0 (2.0, 4.0, 6.0, 8.0, 10.0) and the zeta potential were measured on Malvern Zetasizer Nanoseriesa. A drop of dispersion was spread on a freshly copper grid surface and then the samples were air-dried for TEM analysis. The morphology of graphene was inspected and obtained by TEM (Hitachi H-7650, Chiyoda, Japan). The UV-Vis absorption spectrum was obtained via a Nicolet Evolution 300 UV-Vis spectrometer. Fourier transform infrared (FT-IR) spectra were collected on a Nicolet Avatar-330 spectrometer with 2 cm^−1^ resolution using the KBr pellet technique. Raman spectra were collected with a Renishaw inVia model confocal microscopy Raman spectrometer (Renishaw, Wotton-under-Edge, UK) at an excitation wavelength of 633 nm.

### 2.2. Seed Exposure and Germination

The wheat seeds were purchased from a commercial seed company located in Wuhan, China. The average germination rates of all plant seeds were greater than 90% according to a preliminary study. The seeds were immersed in a 10% sodium hypochlorite solution for 10 min for sterilizing and then they were rinsed three times with deionized water to wipe off the residual disinfectant on the surface [27]. Sterilized seeds were subsequently soaked in deionized water or suspensions supplemented with different concentrations of nanomaterials (125, 250, 500, 1000, and 2000 μg/mL GO or G-NH_2_) overnight at 25 °C. pH values of the test solutions were adjusted to 7 or so. Then, seeds were transferred onto filter papers placed in 100 mm × 15 mm Petri dishes test unit along with 5 mL of corresponding concentration of GO or G-NH_2_, with 10 seeds per dish and 1 cm or larger distance between each seed [28]. The petri dishes were sealed with polyethylene to minimize water evaporation, and then placed in a dark growth chamber under a constant temperature of 25 °C. All solutions and deionized water were renewed every 24 h. Five mL of deionized water was added as the control group. After 24 h and 72 h incubation, when nearly 65% of the control roots were 5-mm long or more [29,30], the germination percentage of seeds was calculated respectively for the total number of seeds (100%) treated under the control condition (deionized water) and under each experimental condition.

### 2.3. Seedlings Investigations

After continuous growth for 9 days in the water and different concentrations of nanoparticles, the *T. aestivum* seedlings were gently cleaned with water to remove the remaining nanomaterials and dry them out with filter paper. Then, the root length and stem length of untreated and treated plants were monitored using calipers. The visible morphology were pictured by Canon EOS 80D. All experiments were performed using a completely randomized design with three replicates per treatment.

### 2.4. Root Structure Observation by Paraffin Section

The root samples were prepared as described above. All the samples were embedded in paraffin and serially cut for experiment use. After deparaffinization in xylene and absolute ethyl alcohol, the slices were eluted separately in a declining ethanol series (95%, 90%, 80% and 70%) for 5 min, and finally in distilled water. The staining was performed as follows. The slices were stained with 0.1% safranine for 1–2 h and washed with distilled water to remove the residue followed by decolorization separately in an ascending ethanol series (50%, 70%, 80% and 90%) for 1 min. Next, the samples were put in fast green stain (0.5%) for 50 s, and then decolorized in absolute ethyl alcohol. Finally, the sections were parched at 60 °C until they turned transparent in xylene for 5 min and then mounted on glass slides with neutral balsam. The paraffin-embedded sections were observed under an optical microscope (Leika, Portland, OR, USA, DCF425).

### 2.5. Morphological Observation by TEM

The root cell morphologies of *T. aestivum* seedlings were observed by TEM (FEI, Hillsboro, OR, USA) to examine the change of root structures after exposure to GO and G-NH_2_ and the uptake of nanomaterials by plant. After growth under hydroponic culture conditions for 9 days in different suspensions (deionized water, 2000 μg/mL GO and G-NH_2_), the roots of *T. aestivum* seedlings were cleaned and cut from the same location, then prefixed in 3.5% glutaraldehyde, washed with 0.1 mol/L pH 7.0 phosphate buffers, and postfixed in 1.0% osmium tetroxide. After fixation, all the treated samples were dehydrated in an ascending ethanol series, and embedded in Spurr’s resin. Finally, the thin sections were excised from the embedded samples using an ultramicrotome equipped with a diamond knife, and the ultrathin sections were mounted on copper grids for TEM examination.

### 2.6. Electrolyte Leakage

The injury of cell membrane was investigated by measuring electrolyte leakage, namely the cytoplasmic leakage. We chose the root as the object of the study, because of the direct contact of the roots with the nanomaterial suspensions. Electrolyte leakage was indicated by the measurement of electrical conductivity as previously reported using a portable conductivity meter (pH/Cond Meter, DDS-11A, Shanghai, China) [19]. After treatment with GO and G-NH_2_ suspensions for 9 days, roots were cut and separated from the seedlings, and then cleaned at least three times with deionized water to remove the contamination on the surface. The seedlings treated with sterilized water were used as the control. Different root samples (100 mg) were placed in a centrifuge tube containing 10 mL of deionized water and incubated at room temperature under 120 rpm for 24 h. The electrical conductivity was detected and labeled as E_1_. Next, the solution was autoclaved at 120 °C for 20 min and the second electrical conductivity was determined, followed by cooling to room temperature, which was labeled as E_2_. The final electrolyte leakage was calculated according to the formula: Final electrolyte leakage (%) = (E_1_/E_2_) × 100.

### 2.7. Statistical Analysis

Data were expressed as mean ± SD of three experiments in triplicate. Error bars represented the standard deviation of the mean. The significant difference was analyzed using the Statistical Package for the Social Sciences (SPSS) version 19.0 software. Statistical comparisons were performed by analysis of variances (ANOVA). The values of *p* (<0.05 and <0.01) were considered to be statistically significant (*) and highly significant (**), respectively.

## 3. Results and Discussion

### 3.1. Characterization of GO and G-NH_2_

**Figure 1:**
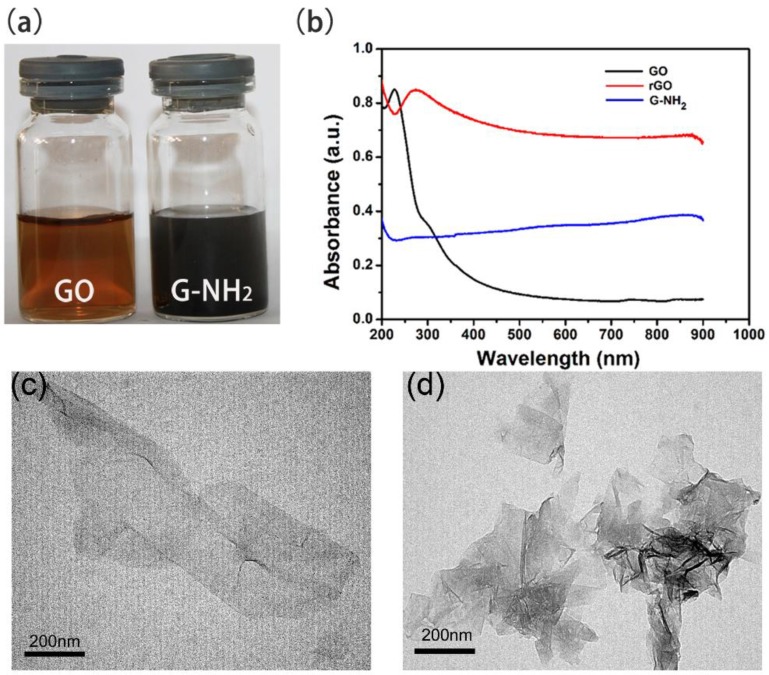
Optical images (**a**); UV-Vis-NIR absorption spectra (**b**) and TEM images of GO (**c**) and G-NH_2_ (**d**) at an individual concentration of 10 mg/L each.

**Figure 2:**
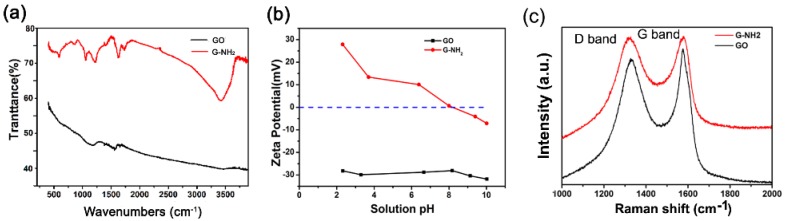
FT-IR spectra (**a**); Zeta potential (**b**) and Raman spectra (**c**) of GO and G-NH_2_ at an individual concentration of 10 mg/L.

GO nanosheets are negatively charged duo to the presence of abundant carboxyl groups on the surface of carbon sheets [31]. Amine-modified graphene oxide was derived from GO sheets by replacing negative carboxyl groups with positive amine groups as previously described [26]. As observed in Figure 1a, there are two kinds of nanomaterials with different colors. The suspension of G-NH_2_ became deep black versus the bright brown of the unfunctionalized GO sample. We further analyzed the characterization of GO and G-NH_2_ using several technologies. UV absorption spectrum was mainly focused on 230 nm because of π-π* transitions of C=C in GO sheets, which was red shifted to 260 nm when GO was reduced to RGO. TEM images showed laminated sheets of GO and G-NH_2_ (Figure 1c,d). From the absorption spectrum, it can be seen that G-NH_2_ displayed strong absorbance in the entire visible and NIR regions. FTIR spectrum investigations showed a characteristic peak at 1573 cm^−1^ and a broad peak in the range of 950–1250 cm^−1^,which were assigned to the N–H and C–N bond stretching, respectively [26]. Zetasizer (S90, Malvern Instruments, Malvern, UK) was applied to analyze the Zeta potential of GO and G-NH_2_ nanomaterials. Figure 2 shows the change curve of potential under various pH conditions ranging from 2.0 to 10.0. G-NH_2_ exhibited positive charge below pH 8.0, while GO was always negative. We further verified the nanomaterials by Raman spectroscopy (Renishaw, Wotton-under-Edge, UK), which is an effective technology for determining the characteristics of graphene-based materials. As shown in Figure 2c, both types of nanomaterials visibly displayed two representative peaks that were commonly called D and G bands at about 1350 and 1592 cm^−1^, respectively [32]. The ratio of D band to G band intensity (*I_D_*/*I_G_*) is about 0.97 in the GO sheets, however, the ratio was increased slightly to 1.0 for G-NH_2_. Similar frequencies and intensity ratio for D and G bands demonstrated that amino groups linked on the surface of GO sheets did not cause any defects in the structural performance of graphene.

### 3.2. Effects of GO and G-NH_2_ on the Growth of Wheat Seedlings

**Figure 3:**
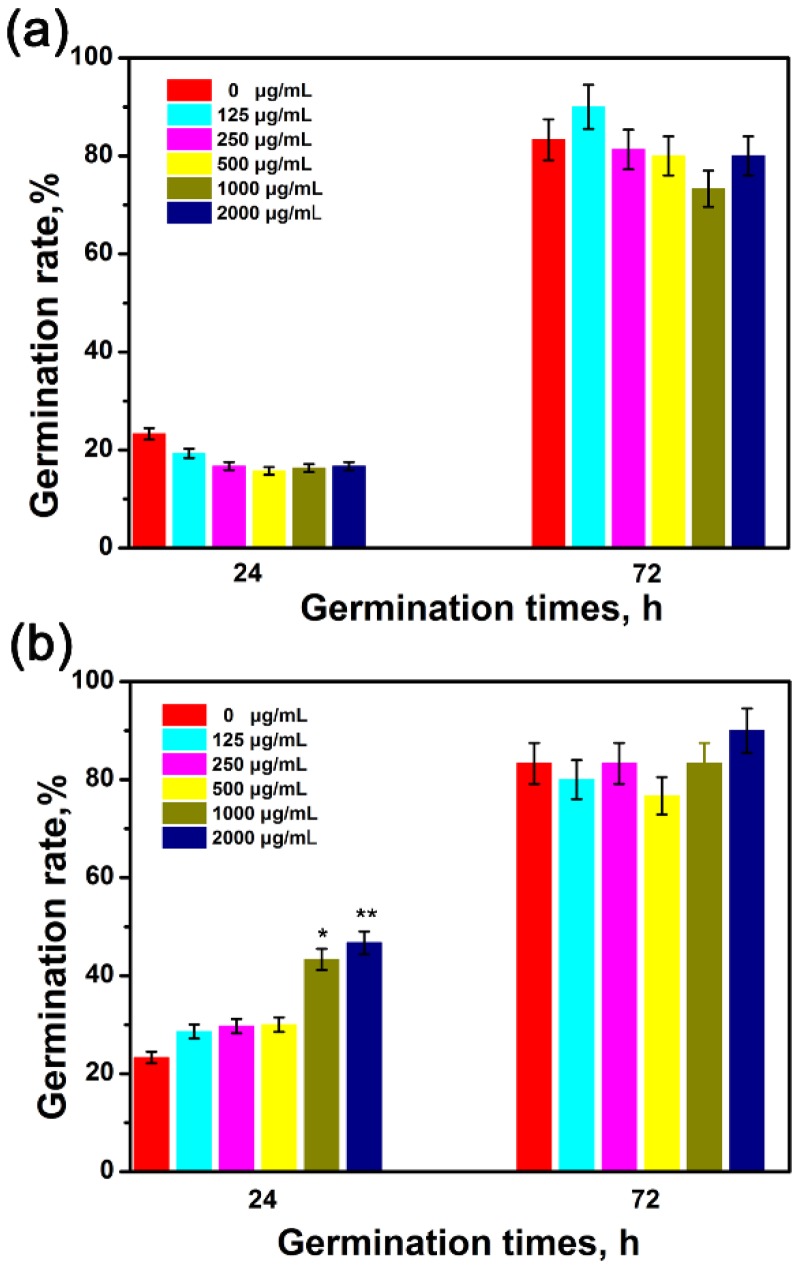
Germination rate of seeds after incubation with different concentrations (0, 125, 250, 500, 1000, 2000 μg/mL) of GO (**a**) and G-NH_2_ (**b**). Results are shown as mean ± SD of measurements of 10 plants per each condition. Where appropriate, statistical significance is indicated by * *p* < 0.05 and ** *p* < 0.01 versus the control.

The germination of wheat seeds and physiology effect of wheat seedling in the presence of GO and G-NH_2_ were evaluated following the hydroponic culture experiments. By far, most studies with engineered nanoparticles have investigated their phytotoxicities on different plants in the concentration range of 10–4000 μg/mL [20,33,34]. According to the USEPA guidelines (USEPA, 1996), the nanoparticle can be reported with the minimal toxicity on test plants if it has no negative effect on seed germination and root growth at the concentration of 2000 μg/mL. Therefore, in this study, 2000 μg/mL was determined to be the highest concentration to examine the phytotoxicity of GO and G-NH_2_ towards wheat. Specifically, sterilized seeds were exposed to deionized water or suspensions with different concentrations of nanomaterials (125, 250, 500, 1000, 2000 μg/mL GO or G-NH_2_) at 25 °C. The germination rate was investigated under 24-h and 72-h exposure conditions, respectively. As shown in Figure 3, seed germination rates were not significantly affected when incubated with GO suspension for 24 h and 72 h. In the case of G-NH_2_, although the wheat seed germination rate was significantly increased under 24-h culture conditions at 1000 and 2000 μg/mL, the final germination rates under all the treatments showed no obvious difference from the 85% germination rate of the control group, as shown in Figure S1. Our results were similar to the effects of amino-functionalized CNT on lettuce seed germination with germination rates of 82–96% [9]. From these results, it can be concluded that both GO and G-NH_2_ were unable to exert toxicity on the germination of seeds. A recent work has indicated that GO at the low concentration range of 400 mg/L dramatically enhanced the seed germination and growth of fava bean plants [35]. Larue et al. (2012) similarly found that the germination rate of wheat was unaffected by exposure to 10–100 mg/L of MWCNT under hydroponics culture, but they were taken up by plant roots and transported to the leaves [36].

**Figure 4:**
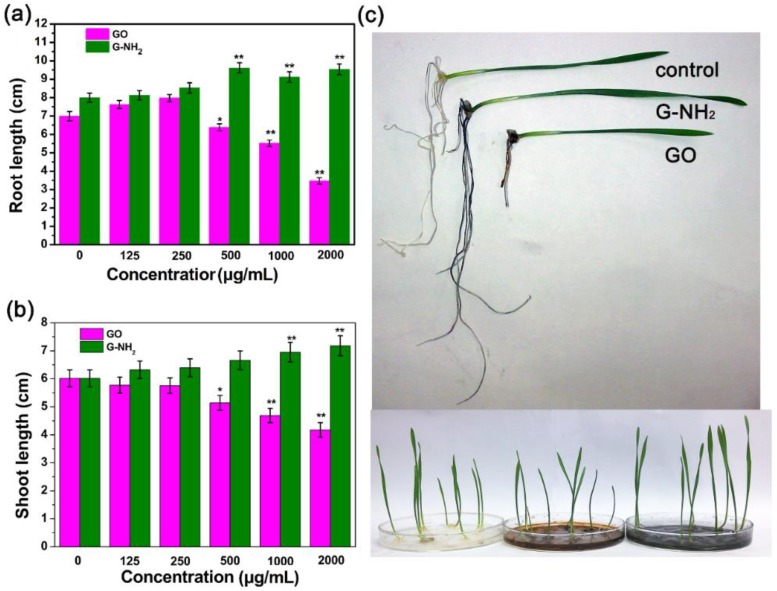
Root length and stem length of seedlings after incubation with different concentrations (0, 125, 250, 500, 1000, 2000 μg/mL) of GO (**a**) and G-NH_2_ (**b**) for 9 days; (**c**) Phenotypes of 9-day-old wheat seedlings grown on medium with water, GO and G-NH_2_. Results are shown as mean ± SD of measurements of 10 plants per each condition. Where appropriate, statistical significance is indicated by * *p* < 0.05, and ** *p* < 0.01.

Phytotoxic phenotypes in plant seedlings caused by nanomaterials include root growth, stem growth, biomass and so on. We further studied the phytotoxicity of functionalized and nonfunctionalized GO sheets. After treatment with various concentrations of GO and G-NH_2_ suspensions for 9 days, the root length and stem length of wheat seedlings were measured. Interestingly, we found that functionalized and non-functionalized GO sheets demonstrated different toxic behaviors. The average root lengths of seedlings exposed to 125, 250, 500, 1000, and 2000 μg/mL GO were 7.62, 7.97, 6.38, 5.51, 3.47 cm, respectively, compared with 6.99 cm of the control sample. The phenotypes of 4- and 9-day-old wheat seedlings exposed to 2000 μg/mL GO and G-NH_2_ are shown in Figure S2 and Figure 4, respectively, indicating that GO sheets can stimulate the root growth at lower concentrations (125 and 250 μg/mL) and the average root lengths were increased by 9.0% and 13.8% in comparison with the untreated wheat seedlings (Figure 4). However, the effects of GO exposure at higher concentrations on root growth were negative, while the average root lengths at 500, 1000 and 2000 μg/mL declined by 8.73%, 21.17% and 50.36%, respectively. These observations at high concentrations were in agreement with Anjum et al. [33], who exposed fava bean to GO at 1600 mg/L and the results found GO results in growth reduction, anti-oxidative enzyme activity (e.g., catalase and ascorbate peroxidase) decease and greater electrolyte leakage, but there was no toxicity below 800 mg/L of GO. The phenomenon was possibly related to the properties of the nanomaterials. A previous study reported that GO sheets can act as a cell growth factor and induce cell division and proliferation at a low dosage [37]. Interestingly, the stem growth was inhibited after incubation with GO suspension and the inhibitory effect was enhanced with increasing concentration, while the stem length of the seedlings was enhanced after exposure to G-NH_2_ (Figure 4b). The root length and stem length were increased by 19.27% and 19.61% under 2000 μg/mL exposure, which can be visually presented in Figure S3 and Figure 4c. The present results are in agreement with the finding of a previous plant study that graphene significantly induced phytotoxicity in vegetable plants after 20 days of exposure in the modified Hoagland medium at 500–2000 μg/mL [19]. Moreover, another observation showed that the combined exposure to GO and PEG 6000 (20%) or NaCl (200 mM) resulted in a more severe toxicity to *Arabidopsis* seedlings, decreasing in fresh weight or root length [38]. Though research on the effects of G-NH_2_ on plants are still rare, it was reported that the G-NH2 has been studied for biomedical applications duo to the less stimulatory action toward platelets and more hemocompatible as compared to GO. In addition, carboxylated (COOH)-MWCNT was found more toxic to biological cells compared to the pristine CNTs, while amino-functionalized CNT was proven to lower the toxicity profiles of CNTs by enhancing clearance [39]. In this paper, G-NH_2_ was nontoxic and enhance the plant growth, we inferred that the result is possibly associated with the enhanced thermally and electrical properties. The introduction of amino could decrease the surface electrical resistivity of GO, having higher electronic conductivity, activating the bioactivity on plant cells [40], improving water and nutrient uptake just like MWCNT [36]. It can be seen that surface functionalization significantly influenced the biological effects of carbon-based nanomaterials.

Rico et al. indicated that engineered nanomaterials (ENMs) exerted obvious toxicity on crop plants in the concentration range from 1000 to 4000 μg/mL [41]. Several recent reports have also indicated that this concentration range was commonly used to assess the impact of nanomaterials on edible plants, including the absorption, translocation and accumulation [42,43,44]. Though the nanomaterials were found to be phytotoxic under different concentrations, it is worth noting that our results showed the toxicity effects of GO on wheat seedlings by restraining root length and seedling growth, but no significant toxicity was observed at lower concentrations (125–500 μg/mL). The better biocompatibility of GO under low concentration is associated with its potential antibacterial application for controlling disease [45].

### 3.3. Plant Structural Observation by TEM

**Figure 5:**
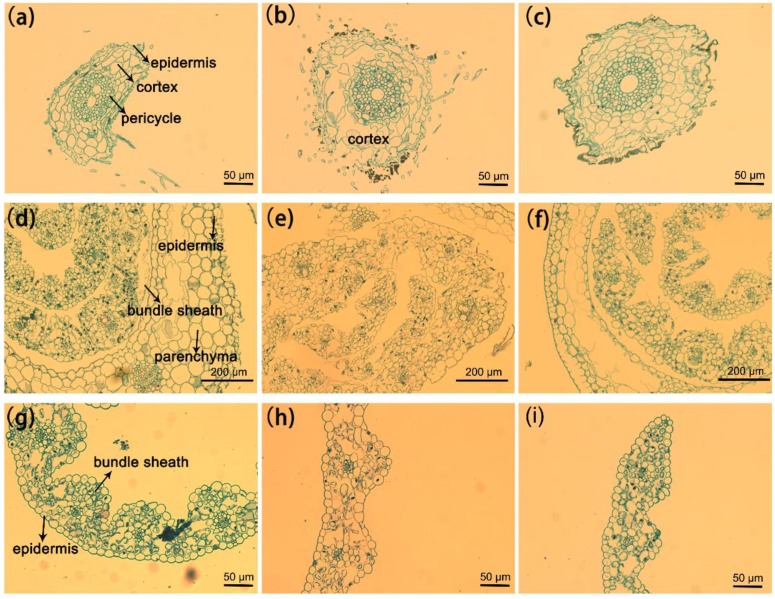
Paraffin section images (cross-sections) of wheat root (**a**–**c**); stem (**d**–**f**) and leaf (**g**–**i**) after incubation with 2000 μg/mL of GO and G-NH_2._ The plants exposed to distilled water were used as control.

The above results indicated that the root and stem were susceptible after direct incubation with GO and G-NH_2_ at a higher concentration. To investigate the structure changes of the wheat plant after incubation with unfunctionalized GO and amine-functionalized GO, root, stem and leaf were cut from treated samples and the cut sections were prepared in the standard paraffin section method. To minimize the damage to plant tissues during the cutting process, all the samples were excised using a blade after sterilization. In the experiment, we selected the high concentration (2000 μg/mL) due to the significant effects compared with the control group. Light-microscopic studies with paraffin section indicated that the plants exposed to concentrations of 2000 mg/L GO showed significant toxicity after 9 days (Figure 5). The root, stem and leaf structure of the untreated plant developed well with the compact tissue including epidermis, cortex and pericycle (Figure 5a). The GO-treated plants displayed significant changes in the root cell and it is worth noting that the epidermal and cortical cells appeared to be strongly disintegrated and loose, with most cells broken, but the pericycle was not affected, and GO was observed on the root surface (Figure 5b). In the case of stems, irregular organizations were observed in the GO treated plant compared with the control without GO exposure, but little impact was found on leaf cells (Figure 5e,h). Based on the cross-sectional images of plant tissues, there was no significant difference between wheat seedlings grown in water and in 2000 mg/L GO-NH_2_ suspension (Figure 5c,f,i).

**Figure 6:**
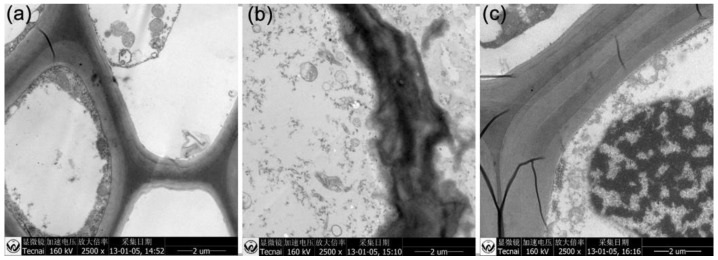
TEM images of the root system of 9-day-old wheat seedlings grown on media without namomaterials (**a**); with GO (**b**) and G-NH_2_ (**c**) after incubation for 9 days.

The present results have proved that GO had the most significantly negative effect on the root system of the wheat plant but GO-NH_2_ did not induce any obvious effect. To investigate the changes in the root cell microstructure and the potential for the graphene uptake by the wheat plant, TEM analysis (FEI, Hillsboro, OR, USA) was performed, which is a powerful technique to evaluate the interaction between nanomaterials and biological samples in uptake, accumulation, and transmission of nanoparticles in terrestrial plants [46]. Figure 6 shows the microscopy images of the root tissues separated from wheat seedlings cultivated under three different conditions for 9 days. In the untreated root, it can be observed that the epidermal cells were fully organized and had the integrated cell wall. In the root cortex of the GO-treated plants, the cell TEM images implied that the root cell walls were significantly fractured and obscure, with irregular folding. However, in the root treated with G-NH_2_, the cell wall was much thicker at the outer periclinal area, in which fine parallel striations were observed. Additionally, TEM images of the cross sections of wheat roots showed the absence of nanomaterials in the present study. It is very likely that GO and GO-NH_2_ may aggregate in the epidermis of wheat root (Figure 5), which is consistent with the investigation of the Cañas group, who have confirmed the presence of nanotube sheets on the root surfaces of crop species, but no visible uptake was observed inside the plant [11]. Although the accumulation of GO and GO-NH_2_ was not observed in the 9-day-old wheat seedlings under hydroculture, there is evidence on the uptake of other carbon nanomaterials by vegetations at various developmental stages [27,30]. A previous study investigated the uptake and translocation of C70 and MWCNTs in the rice plant and found C70 inside the plant root and simultaneously transported via transpiration and the evaporation of water, forming aggregation within the vascular system, while little MWCNTs were absorbed by root cells [46]. Conversely, MWCNTs are accumulated in *Arabidopsis thaliana*, *Onobrychis arenaria*, rice, maize and soybean, changing the biochemical and physiological/morphometric parameters [47,48,49]. Even, under salt stress, MWCNT can enter into broccoli cells with higher accumulation, inducing positive effects on plant growth as consequence of improved water uptake and increased net assimilation of CO_2_. Most importantly, MWCNTs alleviated the abiotic stress by changing in the rigidity and permeability of the root plasma membranes [49]. Thus, further research on the interaction between GO and functionalized GO with the wheat plant, especially the mature plant, is still needed for a comprehensive evaluation of the impact of nanomaterials on the biological system.

### 3.4. Electrolyte Leakage Investigation of Root Cells

**Figure 7:**
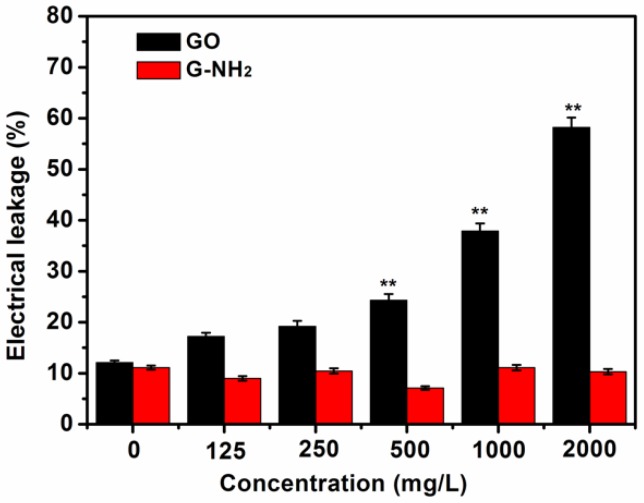
Effect of GO and G-NH_2_ on electrolyte leakage of root cell of wheat seedlings. 9-day-old seedlings grown on media with graphene (0, 125, 250, 500, 1000, and 2000 mg/L) were used for all measurements. Error bars represent standard deviation. Where appropriate, statistical significance is indicated by ** *p* < 0.01.

To further analyze the impact of GO and GO-NH_2_ on the root cells in this experiment, a cell membrane damage assay was conducted by measuring electrolyte leakage of untreated wheat roots and the roots treated with carbon materials. After 9 d of growth under different hydroponic culture conditions, the root samples from the wheat seedlings were cleaned and used to investigate the effect of GO and GO-NH_2_ on cell membrane integrity by examining the electrical conductivity. The higher was the electrolyte leakage, the severer would be cell membrane damage. The GO-treated plants displayed a gradual increase in membrane injury with increasing GO dose. As shown in Figure 7, the levels of electrolyte leakage in the wheat plants treated with low concentrations (125 and 500 mg/L) of GO were not significantly increased in comparison to the control. However, with increasing treatment concentrations, the ion leakage, an indicator for cell membrane damage, was significantly higher than the control (*p* < 0.05), indicating that membrane damage was induced. The plant exposed to the concentration of 2000 mg/L displayed nearly 61% electrical leakage, which is 6.3 times that of the blank plants. In the case of GO-NH_2_, none of the treatments showed significant changes. Carbon-based nanomaterials were previously found to be a dominant cause for generation of ROS in various plants [20,33,50]. A large number of researchers have stated that the accumulation of ROS in biological cells facilitates the electrolyte leakage because ROS can rapidly attack lipids, leading to irreparable membrane damage, followed by cell death [19,51]. It is reported that GO exhibited toxicity effect on terrestrial plants grown hydroponically, including cabbage, tomato, red spinach, and lettuce, by inducing significant damages to root and leaf cell membranes through oxidative stress and reactive oxygen species (ROS) production after 20 days of incubation [11]. GO combined with PVP (20%) or NaCl have been proved by Wang a greater increase in hydrogen peroxide content or membrane ion leakage, decrease in superoxide dismutase activity or catalase activity, and induction of reactive oxygen species production in *Arabidopsis* seedlings [36]. As shown in Figure 5, GO sheets were stuck to the root surface of the seedlings and aggregated together. There is evidence that the carbon-based nanoparticles attached to the root surface inhibited the development of the plant, resulting in significant decreases in root and shoot [11]. It can be speculated that the exposure of the plant roots to GO led to the cytoplasmic leakage due to ROS production, reflecting the injury of the cell membrane, and eventually the cell death.

In the past few years, a number of researchers have pointed out that chemical functionalization of carbon-based nanomaterials can affect the development of plants, resulting in various physiological responses from positive to negative. However, these studies have reached an opposite conclusion about the cytotoxic effect of CNTs. A previous study has reported that functionalized SWCNTs reduced the cytotoxicity [52]. Villagarcia et al. investigated the effects of various surface charges on the physiological response of tomato plants and found that surface characteristics (functional type) were critical for the phytotoxicity of CNTs, i.e., the functionalized CNTs with more negative charge would have better dispersibility and could extremely stimulate the growth of tomato plants [8]. Additionally, amino-functionalized CNT exposure did not significantly influence lettuce seed germination and plant growth. It was noticed that non-functionalized CNT decreased the root and shoot pesticide content while amino-functionalized CNT effects were significantly more modest, likely due to strong competition over adsorption sites on the nanomaterial [9]. To our knowledge, most previous studies have indicated that various engineered nanoparticles (ENPs) had no or a positive effect on plants, biological cells and algae [53,54,55,56], although these nanoparticles, to a certain extent, were shown to cause toxic effects on the plants, such as phytotoxicity and genotoxicity. Especially, TiO_2_ nanoparticles can alleviate the membrane damage indexes in sensitive and tolerant chickpea (*Cicer arietinum* L.) genotypes under cold environmental stress [55]. Other than G-NH_2_, GO sheets were much better dispersed duo to the abundant hydrophilic carboxyl groups on the surface. These sheets induced detrimental actions on the wheat plant, which can be possibly explained by the better uptake of the negatively charged GO sheets by the plant and the production of the ROS species in the cells [19], despite no such observation obtained in our experiment, indicating that more detailed research is needed in this sphere. Additionally, the enhancement effect induced by graphene oxide after amination has not been reported previously and the interactive mechanism remains unclear. Therefore, further research is required to fully understand how surface functionalized groups affect the phytotoxicity on plant biology of GO and functionalized GO.

## 4. Conclusions

As far as we know, little investigation has been done so far on the exposure of functionalized graphene oxide to agricultural and environmental systems. In this study, we evaluated the phytotoxicity of unfunctionalized graphene oxide and amine-functionalized graphene oxide on the wheat plant under various hydroponic culture conditions in terms of seed germination, seedling growth and morphological changes of the wheat plant. Our results indicated that GO inhibited the germination, and G-NH_2_ enhanced it at the beginning (24 h), but neither of them showed any toxicity on the wheat seed germination rate at 72 h. After exposure to GO suspension for 9 days, the growth of *Triticum aestivum* seedlings was significantly restrained, leading to adverse effects on the development of root length, shoot length and biomass as well as morphological damages to root cells. However, G-NH_2_ was found to facilitate seedling growth. Additionally, in the present experiment, neither of the two-graphene forms was observed to aggregate in the root cells. In the future, more studies are needed to clarify the phytotoxicity and how GO and G-NH_2_ exert toxicity on wheat or other higher plants in the biological system.

## Supplementary Materials

The supplementary materials are available online.
