# Supplementary material for: Various Physiological Response to Graphene Oxide and Amine-Functionalized Graphene Oxide in Wheat (Triticum aestivum)
Source: Molecules. 2018 May 7;23(5):1104. doi: 10.3390/molecules23051104 (PMC6100068; doi:10.3390/molecules23051104)
Supplement: Supplementary file 1 [file molecules-23-01104-s001.pdf]

Various Physiological Response to Graphene Oxide and Amine-Functionalized Graphene Oxide in Wheat (*Triticum aestivum*)

Juanni Chen, Liang Yang, Shili Li and Wei Ding *

Laboratory of Natural Product Pesticide, College of Plant protection, Southwest University, Chongqing 400715, China; jemmy@swu.edu.cn (J.C.); ylwzling@163.com (L.Y.); lsl203lst@163.com (S.L.)
* Correspondence: dingw@swu.edu.cn; Tel.: +86-23-6825-0953; Fax: +86-23-6825-0953

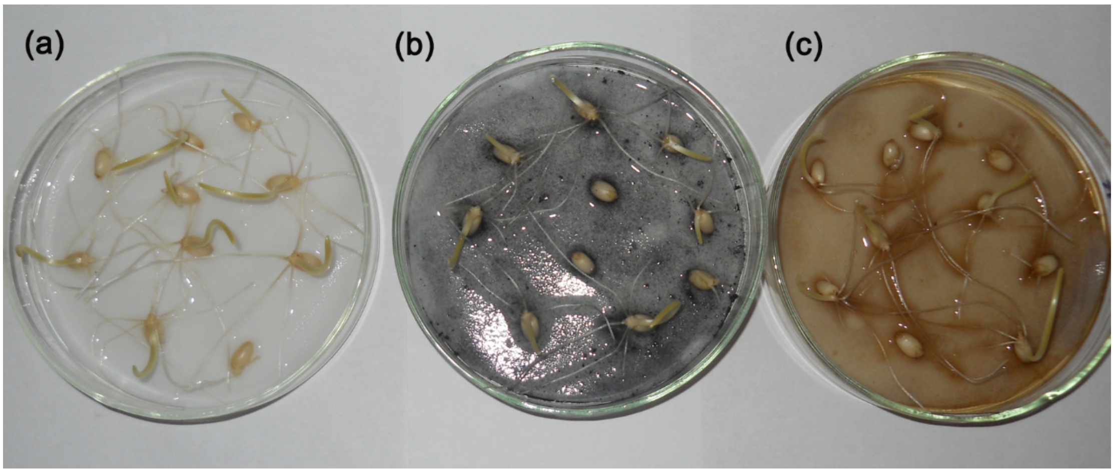

Figure S1. Phenotypes of wheat seed germination after incubation with (**a**) water, 2000 μg/mL of (**b**) GO and (**c**) G-NH_2 for 72 h.

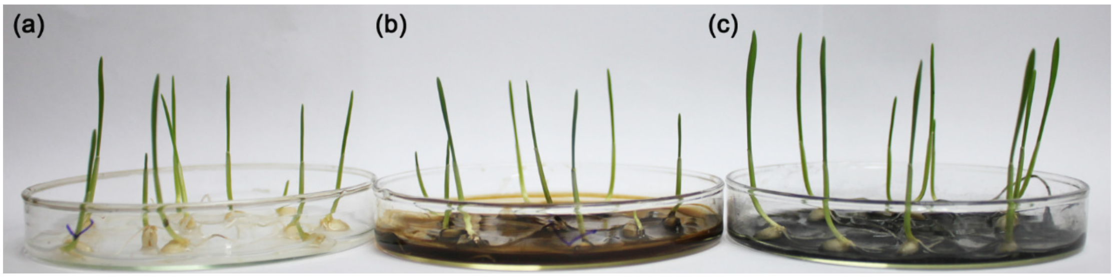

Figure S2. Phenotypes of 4-day-old wheat seedlings grown on medium with (**a**) water, 2000 μg/mL of (**b**) GO and (**c**) G-NH_2.

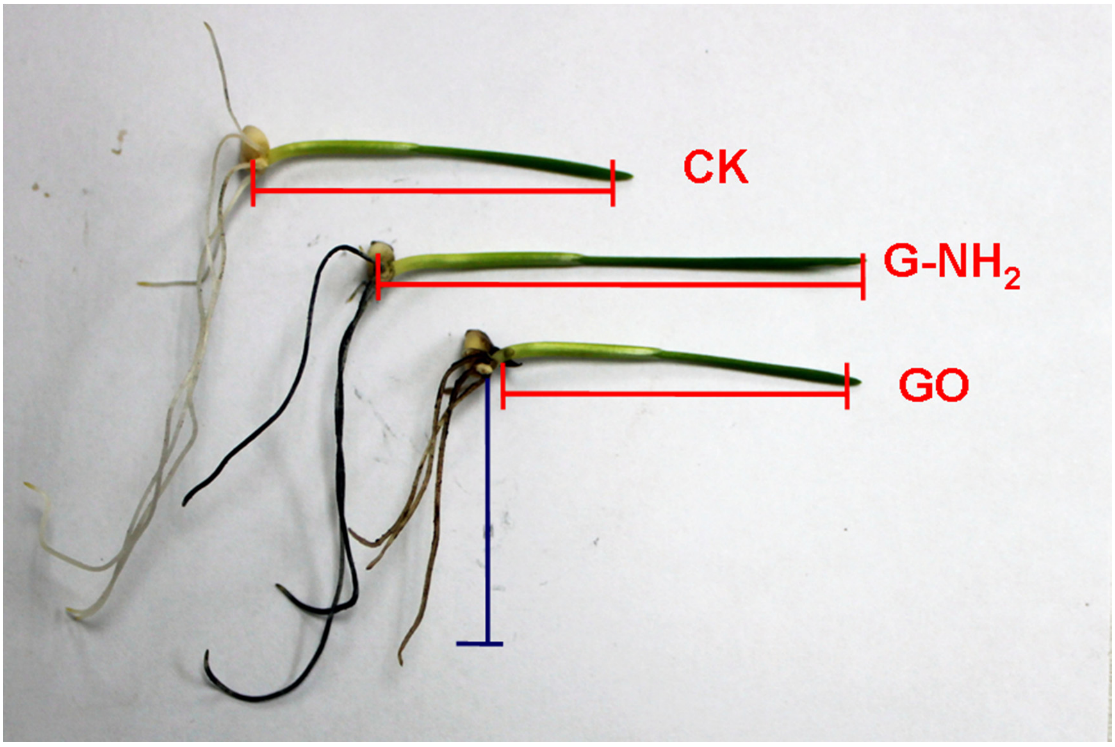

Figure S3. Phenotypes of 4-day-old wheat seedlings grown on medium with water (CK), 200 0μg/mL ofGO and G-NH_2.
